# IA期肺腺癌病理高危因素及术后辅助化疗研究进展

**DOI:** 10.3779/j.issn.1009-3419.2022.101.30

**Published:** 2022-08-20

**Authors:** 晨 沈, 文涛 方

**Affiliations:** 200030 上海，上海市胸科医院，上海交通大学医学院附属胸科医院胸外科 Department of Thoracic Surgery, Shanghai Chest Hospital, Shanghai Jiao Tong University School of Medicine, Shanghai 200030, China

**Keywords:** 肺肿瘤, 病理学, 预后, 辅助化疗, Lung neoplasms, Pathology, Prognosis, Adjuvant chemotherapy

## Abstract

早期非小细胞肺癌患者的生存率有待提高。含实体或微乳头成分、伴脉管侵犯或伴气道播散的浸润性腺癌患者属于复发风险高危人群，需要探索包括根治性手术切除在内的系统治疗方案。而现有指南不推荐IA期患者行术后辅助化疗。本文将综述上述病理高危因素在IA期肺腺癌患者中的研究进展以及辅助化疗在IA期复发风险高危人群中的作用。

## Abstract (en)

The survival rate needs to be improved in early stage non-small cell lung cancer patients. The risk of recurrence is relatively high in invasive adenocarcinoma patients with a solid or micropapillary component, lymphovascular invasion or tumor spread through air spaces. Systemic treatment options including radical surgical resection should be explored for this population. Adjuvant chemotherapy is not recommended for patients in stage IA in current guidelines. This article is a review on the research progress of the above pathological high-risk factors and the role of adjuvant chemotherapy in patients with pathological high-risk factors in stage IA lung adenocarcinoma.

## 1 前言

全球范围内肺癌发病率和死亡率居高不下，肺癌是我国疾病负担最重的肿瘤性疾病^[1, 2]^。早诊早治是提高肺癌生存率的关键。非小细胞肺癌（non-small cell lung caner, NSCLC）是肺癌的主要类型，其中肺腺癌占比逐步上升。依据第八版肿瘤原发灶-淋巴结-转移（tumor-node-metastasis, TNM）分期，IA期NSCLC患者接受根治性手术切除后预后较好，其中IA1期5年总生存率（overall survival, OS）超过90%。然而随着T分期的增加，患者生存率逐步下降，IA3期5年OS仅为77%^[3]^。既往研究显示IA期患者术后辅助化疗（adjuvant chemotherapy, ACT）无生存获益^[4]^，因此目前各指南均不推荐IA期行ACT^[5-7]^。除了T分期外，组织学亚型、脉管侵犯（lymphovascular invasion, LVI）、气道播散（spread through air spaces, STAS）等也是影响肺腺癌患者预后的重要因素，本文将综述上述病理高危因素在IA期中的研究进展，并探讨ACT在IA期含病理高危因素患者中的作用。

## 2 ACT

1995年NSCLC合作小组进行了一项包含了14项临床试验共4, 357例Ⅰ期-Ⅲ期患者的*meta*分析^[8]^，结果显示对比单纯手术，ACT降低了13%的死亡风险，尽管差异未达到显著性水平（*P*=0.08）。在这之后陆续开展的5项前瞻性临床研究被纳入肺癌顺铂辅助化疗评估（lung adjuvant cisplatin evaluation, LACE）合作小组的*meta*分析^[4]^中，其结果显示化疗组预后明显改善（HR=0.89, *P*=0.005），5年OS提高5.4%。

LACE研究按照分期分层后显示IA期患者化疗并不能改善生存（HR=1.41, 95%CI: 0.96-2.09），近期的回顾性研究和*meta*分析^[9, 10]^也得出了相同的结论。对于Ⅰ期患者，目前指南仅推荐IB期经筛选后的部分高危患者行ACT^[5-7]^。由此在IA期中，能否像IB期那样找到部分可从ACT中获益的人群，是本文的讨论重点之一。

## 3 病理高危因素

肺腺癌患者即使是同一分期，受到病理高危因素和驱动基因的影响，预后也存在较大的异质性，如浸润性腺癌主要亚型就是独立于TNM分期影响预后的重要因素^[11]^，其中实体和微乳头亚型被定义为高级别模式^[12]^。另外，筛状和融合腺体是非传统的复杂腺体模式，研究发现以复杂腺体为主的患者预后与实体和微乳头型患者相当^[13-15]^，且在一些主要亚型中，复杂腺体成分的出现（即占比≥5%）提示预后不良^[16-19]^。

浸润性腺癌的变体包括黏液腺癌、胶样、胎儿型和肠型腺癌，由于发病率较低，生存数据有限。以往研究认为相较于非黏液腺癌，浸润性黏液腺癌患者预后更差^[20, 21]^，然而越来越多的研究指出，黏液腺癌患者虽然肺内复发率更高^[22, 23]^，但其预后并不差于，甚至优于非黏液腺癌^[24-27]^；胶样腺癌的临床行为较为惰性，其预后与分期密切相关^[28-31]^；胎儿型腺癌分为高级别和低级别两类，高级别胎儿型患者预后较差^[32, 33]^，其生存率与微乳头型患者相似^[34]^；肠型腺癌具有侵袭性，在确诊时往往分期较高，因此预后较差^[35-37]^。

LVI也称脉管癌栓，指肿瘤侵犯血管或淋巴管，在20世纪60年代就有学者发现肺癌患者伴LVI与预后不良有关^[38]^。有研究^[39]^指出对于Ⅰ期-Ⅲ期NSCLC伴LVI的患者，复发风险将增加1.78倍，5年无复发生存率（recurrence-free survival, RFS）显著降低（52.6% *vs* 87.5%, *P* < 0.001）。

STAS的概念在几十年前已被提出^[40]^，2015年世界卫生组织官方发布了它的定义：微乳头簇状、实性癌巢或单个癌细胞扩散至肿瘤边缘外肺实质的气道内^[41]^。研究^[42]^认为不管是在早期还是晚期的肺腺癌中，STAS均与较差的RFS和OS相关。

表皮生长因子受体（epidermal growth factor receptor, *EGFR*）突变是肺腺癌中一个最主要的驱动基因变异亚型。根据ADJUVANT研究的结果，与非*EGFR*突变型患者相比，*EGFR*突变型发生脑转移和骨转移的风险更高^[43, 44]^，但是在可手术肺腺癌患者中*EGFR*突变是否会增加复发风险，影响总体预后，目前尚无定论。部分学者认为*EGFR*突变型患者预后反而更好^[45-47]^，这些研究未按组织学主要亚型进行分层，事实上分化程度较高的亚型中*EGFR*突变频率反而更高^[48-51]^。Ito等^[52]^在对亚型和分期分层后发现在Ⅰ期以实体或微乳头为主（solid/micropapillary-predominant, S/MPP）和IIA期-IIIA期患者中，*EGFR*突变型5年RFS明显更差（49.6% *vs* 75.6%, *P* < 0.01）。在现有针对IA期的研究中尚未看到明确的证据证明*EGFR*突变状态对预后的显著影响^[53-55]^，因此尽管分子病理层面更贴近肿瘤发生发展的本质，目前对上述组织病理层面高危因素的讨论仍十分重要。

以下将具体论述组织学亚型、LVI和STAS对IA期患者预后的影响。另外有研究^[56-58]^表明核等级、有丝分裂等级等因素同样与预后相关，不在本文的讨论范围。

### 3.1 组织学亚型

#### 3.1.1 组织学主要亚型

在IA期中，相较于其他主要亚型，实体或微乳头型为主的患者预后更差，可能由于样本量的限制，二者差异并不明显^[59, 60]^，因此通常将二者合并为一组进行分析。Ito等^[61]^的数据显示IA期S/MPP患者5年RFS显著低于其他主要亚型（83.3% *vs* 95%, *P* < 0.05）。

#### 3.1.2 微乳头成分（micropapillary component, MIP）

除主要亚型外含有的次要亚型（micropapillary-minor, MPM）成分，以及各亚型所占百分比，尤其是实体和微乳头等高级别模式的百分比同样需要关注。学者一般依据是否含有高级别模式（即占比≥5%）或高级别模式所占百分比进行研究。

IA期患者含有MIP是预后的危险因素^[62, 63]^，文献多聚焦于MIP百分比对预后的影响。MIP仅为5%时可能不足以影响患者预后，如Su等^[64]^发现在CT表现为纯实性、肿瘤大小≤2 cm的肺腺癌患者中，MIP < 5%和MIP=5%预后无差异（5年RFS：79.9% *vs* 80.2%，*P*=0.057）。MIP占比上升后，其对预后的影响开始明显；当MIP占比达到更高数值时，后续比例继续增加观察到的组间生存差异可能会变得不太显著。Tsubokawa等^[65]^发现MIP≥30%和MIP为5%-25%相较于MIP < 5%的患者5年无病生存率（disease-free survival, DFS）显著更差，但前两者之间差异不明显（48.1% *vs* 76%, *P*=0.213）；同样，Su等^[64]^研究中MIP≥25%和MIP为10%-20%的患者相比预后无差异，但都明显差于MIP≤5%的患者。

当仅讨论为MPM的MIP时，其对预后的影响存在争议。Ito等^[61]^发现IA期中MPM对比不含微乳头成分（micropapillary-negative, MPN）的患者5年RFS差异不明显（90.9% *vs* 96.4%, *P*=0.207），同样在Huang等^[60]^的研究中MPM与MPN患者的DFS生存曲线几乎重合，这可能是因为MPM群体包含了较多MIP仅为5%的患者。但在Yanagawa等^[66]^的研究中，Ⅰ期MPM患者RFS明显差于不含微乳头且不含实体成分（solid component, SOL）的患者（HR=5.49, *P* < 0.001）。

#### 3.1.3 SOL

一般认为IA期含有SOL则预后较差^[62]^，尽管在Su等^[64]^研究中SOL≥10%对比SOL≤5%在单因素分析中RFS和OS均差异显著，在多因素分析中SOL≥10%是OS的独立危险因素（*P*=0.042），但与RFS无关（*P*=0.124）。这可能是因为在讨论SOL时存在MIP的干扰。因此为了在研究某一成分时消除混杂成分的干扰，也有学者将患者如下分组：A组为含MIP，B组为含SOL但不含MIP，C组为含乳头成分（acinar component, ACI）但不含MIP和SOL，D组含腺泡成分但不含MIP、SOL和ACI，E组为仅含伏壁成分。Wang等^[67]^发现在Ⅰ期患者中仅A组（HR=10.784, *P*=0.002）、B组（HR=5.428, *P*=0.022）是RFS的独立危险因素，C组、D组对比E组预后差异不显著。Yanagawa等^[66]^采用类似上述分组方法发现在排除掉S/MPP后，A组与B组预后无差异（*P*=0.53），但都显著差于E组。鉴于实体和微乳头成分（solid and micropapillary component, S+MP）对预后影响差别不大，近期研究倾向于合并这两种高级别模式，将S+MP百分比相加进行分析。

#### 3.1.4 高级别模式百分比

Choi等^[68]^认为IA期中S+MP为5%时，患者5年RFS就显著差于不含S+MP者（73% *vs* 93%, *P* < 0.001）。与MIP类似，S+MP所占百分比达到一定数值后，继续增加可能不能拉开后续组间的生存差异。Huang等^[60]^发现S+MP≥40%对比S+MP为10%-35%（51.5% *vs* 72.7%, *P* < 0.001）和S+MP≤5%（51.5% *vs* 85%, *P* < 0.001），5年DFS均差异显著，而Peng等^[62]^则发现S+MP≥55%和S+MP为5%-50%的预后无差异（*P*=0.177）。

国际肺癌研究协会病理委员会将284例Ⅰ期肺腺癌患者数据作为训练集，通过受试者特征工作曲线（receiver operating characteristic curve, ROC）比较不同模型，得到了将组织学主要亚型和高级别模式（实体、微乳头、复杂腺体成分）百分比作为预后分级依据的最佳模型：1级（高分化）为伏壁型为主且高级别模式 < 20%，2级（中分化）为腺泡或乳头型为主且高级别模式 < 20%，一旦高级别模式≥20%则为3级（低分化）^[69]^。该模型在验证集和训练集中的曲线下面积（area under curve, AUC）和重现性都较好，后续文献^[58, 70]^也证明了该模型的可行性。然而其模型最佳AUC仅略高于0.7，说明需要更多临床病理信息的发掘和纳入以改善模型。

### 3.2 LVI

是否伴LVI是影响IA期患者预后的重要因素^[71-74]^，IA期LVI阳性率多介于10%-20%之间。伴LVI的IA1期和IA2期患者5年生存率在85%以上，IA3期则低于70%^[75-79]^，事实上这样的数值已接近IB期患者整体的生存率。在Tsuchiya^[80]^、Kudo^[77]^、Wang^[81]^分别针对第五版、第七版、第八版TNM分期的研究中也证明了IA期伴LVI与IB期不伴LVI患者预后是相似的。LVI与脏层胸膜侵犯（visceral pleural invasion, VPI）都被认为是侵入性的生长模式，二者常被一同讨论。对于肿瘤大小≤3 cm的Ⅰ期NSCLC患者，Moon等^[82]^发现RFS的独立危险因素是LVI而不是VPI（LVI: *P*=0.006; VPI: *P*=0.106），而Wang等^[81]^则认为LVI和VPI都是RFS的独立危险因素，并发现将LVI和VPI一同纳入进行预后分层后能更好地区分患者的预后。

### 3.3 STAS

在IA期中，STAS同样是预后的危险因素，有26.7%-32.3%的患者表现为STAS，伴STAS的根治性手术切除患者5年DFS为70%-80%^[83, 84]^。与LVI类似，许多学者发现IA期伴STAS患者的预后接近IB期整体患者^[85-87]^，并进行了进一步的分层讨论。Dai等^[86]^发现IA期伴STAS与IB期患者预后的统计学差异处于临界值（*P*=0.091），但当剔除掉T1a和T1b后，T1c伴STAS与IB期患者生存曲线则几乎重合（*P*=0.842）。Han等^[87]^通过评估播散气道与肿瘤边缘的距离将STAS分为近者为Ⅰ级，远者为Ⅱ级，发现IA期患者仅当STAS为Ⅱ级时预后方与IB期相当。

综上所述，T分期、组织学亚型、LVI、STAS等因素共同影响IA期患者的预后，因此为了综合评判这些因素的影响及影响大小，文献常建立列线图这一复发风险评分模型，用于评价模型区分度的C指数报道在0.667-0.773之间^[53, 60, 73]^。依据列线图得到患者评分后可进行预后分层，找出复发风险高危人群，从而进行后续的研究。

## 4 辅助化疗在IA期含病理高危因素患者中的作用

既往研究显示ACT在IA期根治性切除患者中治疗效果有限，一定程度上是因为临床试验纳入了部分预后较好、可能不需要治疗的患者。因此需要筛选出含病理高危因素的复发风险高危人群后再探究ACT的作用。Pathak等^[88]^使用美国国家癌症数据库进行了一项包含50, 814例IA期-IIB期患者的回顾性队列研究发现，当肿瘤大小≤3 cm时，即使是肿瘤分化较差（HR=1.02, *P*=0.81）或伴LVI（HR=0.88, *P*=0.35）的患者，化疗也不能显著改善生存。本文作者一项未发表的针对SOL≥5%或MIP≥5%或伴LVI的1, 406例IA期肺腺癌患者的回顾性研究同样发现，倾向性评分匹配后ACT无显著生存获益（HR=0.83, *P*=0.625）。

然而也有学者得到了不一样的结果。Sasada等^[89]^发现在IA期排除掉浸润前病变和伏壁型为主的浸润性腺癌后，化疗组5年OS优于对照组（95% *vs* 81.1%, *P*=0.04）。但该研究并未在IA期患者中进行配比分析，其对照组中亚肺叶切除明显多于化疗组（*P* < 0.001），而有研究表明楔形切除是伴STAS患者预后的独立危险因素^[84]^，当MIP超过5%时，即使是肺段切除其预后也会显著差于肺叶切除^[64]^。另外的一些研究则认为IA期中以微乳头为主^[54]^，或是肿瘤低分化^[90]^是能从ACT中获益的群体，然而这些研究同样存在没有进行配比分析控制混杂因素、样本量较小等缺陷。Wang等^[91]^开展了包含1, 595例IA期NSCLC患者的大样本回顾性研究，发现IA期和IB期伴LVI的患者均为ACT的获益人群，但该研究进行配比分析时组织学类型仅按照腺癌和非腺癌进行平衡，未涉及浸润性腺癌的主要亚型尤其是高级别模式，尽管LVI与主要亚型密切相关^[65, 68, 71]^。

也有学者通过建立模型进行预后分层后来评估辅助化疗的作用。Qian等^[92]^通过研究4, 606例Ⅰ期肺腺癌患者数据，选择与RFS密切相关的年龄、性别、肿瘤大小、组织学主要亚型、是否伴LVI、是否伴VPI共6个因素建立列线图，根据评分将患者分为低危、中危和高危组，结果表明化疗仅在高危组中显示生存获益（*P*=0.041, 6）。但其所划分的高危组中IA期仅占12%，且未说明这14例患者的特征，无法得知患者是由于列线图中最大评分值最高的三个因素——组织学主要亚型、年龄、肿瘤大小中的哪几个因素而归类至高危组。对于这类纳入年龄因素的模型，在判断高龄患者是否行ACT时需要综合评估患者复发风险、功能状态和可能的生存获益等因素，尽管ACT的疗效不因年龄而改变^[93-95]^。

## 5 总结

实体或微乳头亚型、LVI和STAS等均是IA期肺腺癌患者独立于TNM分期的病理高危因素，单纯手术治疗对含病理高危因素的患者作用有限，需要探索合适的系统治疗方案。ACT对这部分患者的作用目前仍有争议，已有的证据并不支持术后化疗。鉴于*EGFR*突变型提示预后不良，而ADAURA研究^[96]^显示IB期-IIIA期携带*EGFR*敏感突变的患者可从术后辅助第三代EGFR酪氨酸激酶抑制剂（tyrosine kinase inhibitor, TKI）中获益，因此第三代TKI在IA期*EGFR*敏感突变型患者，尤其是复发高危人群中的疗效值得探索和期待。如何安全、有效、精准地进行术后辅助治疗以改善IA期复发高危人群的生存，是未来需要解决的问题。
